# A novel MRI compatible mouse fracture model to characterize and monitor bone regeneration and tissue composition

**DOI:** 10.1038/s41598-020-73301-y

**Published:** 2020-10-01

**Authors:** Nina Schmitz, Melanie Timmen, Katharina Kostka, Verena Hoerr, Christian Schwarz, Cornelius Faber, Uwe Hansen, Romano Matthys, Michael J. Raschke, Richard Stange

**Affiliations:** 1 Department of Trauma, Hand and Reconstructive Surgery, University Hospital Muenster, Münster, Germany; 2 Department of Regenerative Musculoskeletal Medicine, Institute of Musculoskeletal Medicine, University Muenster, Albert-Schweitzer-Campus 1, W1, 48149 Münster, Germany; 3 Translational Research Imaging Center, Clinic of Radiology, University Hospital Muenster, Münster, Germany; 4 RISystem AG, Landquart, Switzerland

**Keywords:** Medical research, Translational research

## Abstract

Over the last years, murine in vivo magnetic resonance imaging (MRI) contributed to a new understanding of tissue composition, regeneration and diseases. Due to artefacts generated by the currently used metal implants, MRI is limited in fracture healing research so far. In this study, we investigated a novel MRI-compatible, ceramic intramedullary fracture implant during bone regeneration in mice. Three-point-bending revealed a higher stiffness of the ceramic material compared to the metal implants. Electron microscopy displayed a rough surface of the ceramic implant that was comparable to standard metal devices and allowed cell attachment and growth of osteoblastic cells. MicroCT-imaging illustrated the development of the callus around the fracture site indicating a regular progressing healing process when using the novel implant. In MRI, different callus tissues and the implant could clearly be distinguished from each other without any artefacts. Monitoring fracture healing using MRI-compatible implants will improve our knowledge of callus tissue regeneration by 3D insights longitudinal in the same living organism, which might also help to reduce the consumption of animals for future fracture healing studies, significantly. Finally, this study may be translated into clinical application to improve our knowledge about human bone regeneration.

## Introduction

Fracture healing is a frequently investigated process in basic research using different small animal models. In clinical studies, advanced imaging technologies like positron-emission-tomography combined with computed-tomography (PET/CT), microcomputer-tomography (µCT) and magnetic-resonance-imaging (MRI) are used frequently and contribute to a new understanding of bone tissue composition, regeneration and disease^1–3^. Technical progress with regard to increased resolution and the development of contrast agents and labeling techniques provides new insights also in small animal analysis of musculoskeletal tissues, now^4–6^. Zachos et al.^7^, Malaval et al.^8^ and Taha et al.^9^ used non-invasive MRI and a bone quantification procedure to follow bone regeneration and healing in rodents. They concluded that MRI is suitable to analyze bone structures providing qualitative and quantitative data without radiation exposure. Recently, in a rat patellar tendon-healing model^10^, we showed that biomechanical stability correlated well with the development of new vessels in the injured tendon monitored and quantified by MRI^11^. We could demonstrate, that vessel size and distribution measured by MRI correlated significantly with immunohistochemical vessel staining over time.

**Figure 1:**
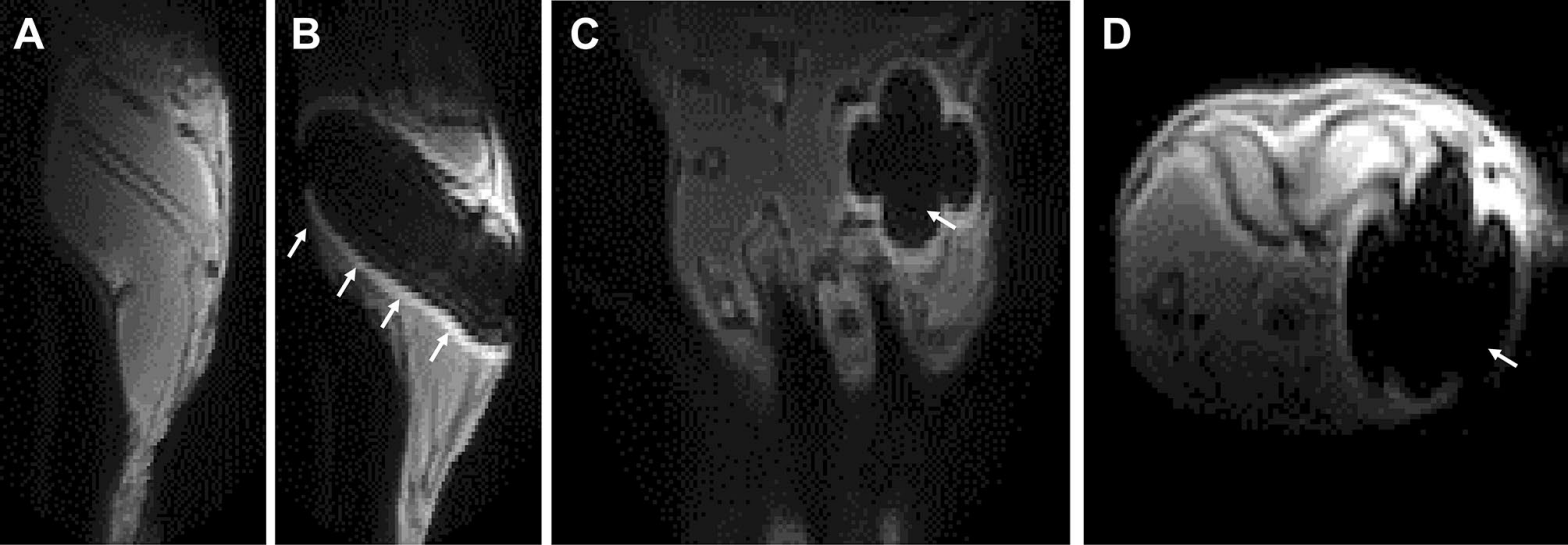
In vivo 3D UTE MRI (Magnetic Resonance Imaging) at 9.4 T of a murine femur without implant (**A**) and with a metal implant (**B**–**D**, gauge needle, n = 1). Pronounced artefacts (white arrows) were generated by the metal implant in sagittal (**B**), coronal (**C**) and axial (**D**) MR images.

Biomechanical surroundings and stabilization techniques are known to play an important role during fracture healing^12,13^. Therefore, various fixation devices have been developed and are used for stabilization of fractures in rodents. This includes external fixator, internal rigid or flexible plates or different intramedullary nails that can be fixed by pins or locked within the cortical bone^14–19^. Biomechanical analyses, histomorphometry and immunohistochemistry can be well performed with these devices. However, all these commonly used devices in rodents as well as most of human implants consist of metal like stainless steel or titanium and therefore are not suitable for MRI due to the artefacts generated by metal (Fig. 1)^20^. Due to the impact of stabilization and biomechanical environment on bone regeneration, few MRI studies of bone healing have been performed without stabilization with conventional implants^9,21^.

Utilizing MRI for fracture healing characterization would help to analyze and quantify e.g. vascularization as well as surrounding muscle and cartilage tissue in one animal continuously in situ, providing 3D information and using much lower amounts of animals at the same time. In our group, the MRI technique was also used successfully to monitor fracture healing in a tibial and femoral rat model using a PLLA pin (poly-l-lactide-pin) to stabilize the fracture intramedullary^22^. Recently, Jin et al*.* (2016) used polyetheretherketone (PEEK) threaded rods in a rat femur fracture model and proposed the feasibility of MRI analysis without metal artefacts as an important method^23^. Haffner-Luntzer et al. have demonstrated in detail the monitoring of longitudinally bone healing by MRI^24^ using a ceramic external fixator in mice (RISystems AG, Landquart, Switzerland). However, they both performed an open osteotomy approach to the femur, which provides a good reproducibility of the bone defect and reflect a more elective bone healing model, lacking the typical epiphenomenon’s of trauma like fracture hematoma or soft tissue damage. We developed a fracture model stabilized with an intramedullary nail in mice and rats over a long time with different applications^25–27^. In contrast to an osteotomy model stabilized with an external fixator, this model simulates a real fracture situation and a frequently used procedure of stabilization for long bones in trauma surgery offering less invasive surgery and fast weight carrying capacity^19^.

We were recently involved in developing a prototype ceramic MouseScrew (now provided by RISystems, Landquart, Switzerland) based on an approved metal implant. In the present study, we investigated this novel intramedullary ceramic nail for fracture stabilization and monitoring of bone healing by MRI in a proof-of-principle approach.

## Results

### Development of the ceramic MouseScrew

**Figure 2:**
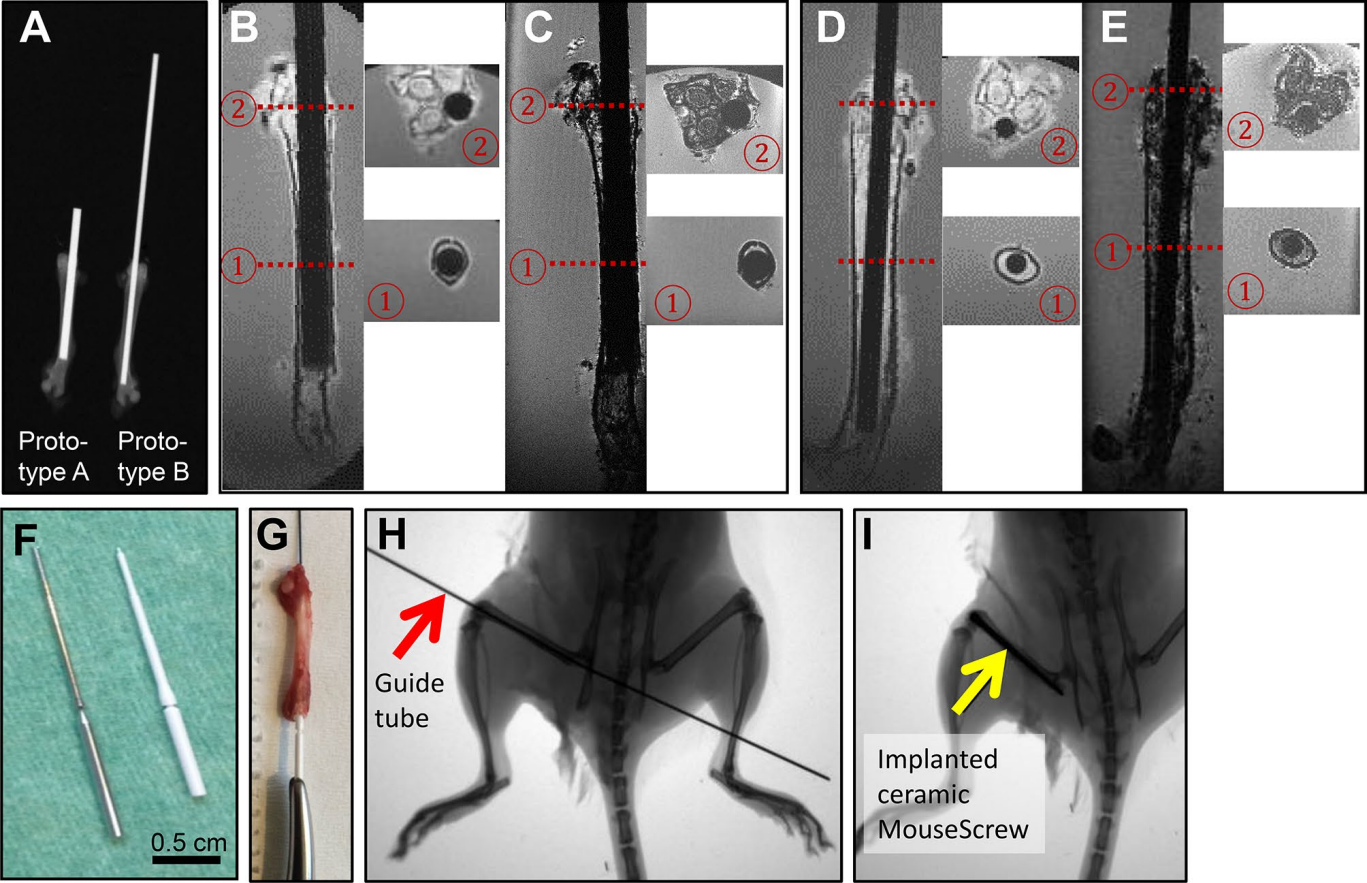
Development of the ceramic MouseScrew. (**A**) Testing of prototypes of different diameters within a dissected mouse femur (ex vivo X-ray). (**B**–**E**) Testing of prototypes in a dissected femur by ex vivo MRI: 3D FLASH images (**C**,**E**) and 3D UTE images of the femurs are shown in coronal view and cross sections of the proximal femur. (**F**) Final shape of the ceramic MouseScrew (right) compared to the metal MouseScrew (left). (**G**–**I**) Testing of implantation of the ceramic MouseScrew in a dissected femur (**G**) and in vivo (X-ray; (**H**) with guide tube, red arrow), (**I**) leg with implant, yellow arrow).

To optimize anatomical and material parameters of the device, we implanted prototypes of different diameter and ceramic (zirconium dioxide, ZrO_2_) material into a dissected mouse femur (Fig. 2A, X-ray) in order to determine the right diameter, length and type of ceramic for usage in adult mice. We additionally examined the material device implanted in a mouse femur by ex vivo MRI (Fig. 2B–E) using 3D UTE and 3D gradient-echo (FLASH) sequences. With both methods, contours of the implant could be separated sharply from the surrounding bone tissue. The prototype of the ceramic MouseScrew with the dimensions based on our preliminary testing and on the shape of the metal MouseScrew is shown in Fig. 2F (length:17.20 mm, thickness: 0.05–0.07 mm). The procedure of implantation was tested and optimized in cooperation with the manufacturer. The implantation, performed equivalent to the metal MouseScrew using a guide tube to which the screw is connected and inserted into the bone, is demonstrated ex vivo (Fig. 2G) and in vivo (Fig. 2H,I, X-ray).

### Surface structure and biocompatibility

**Figure 3:**
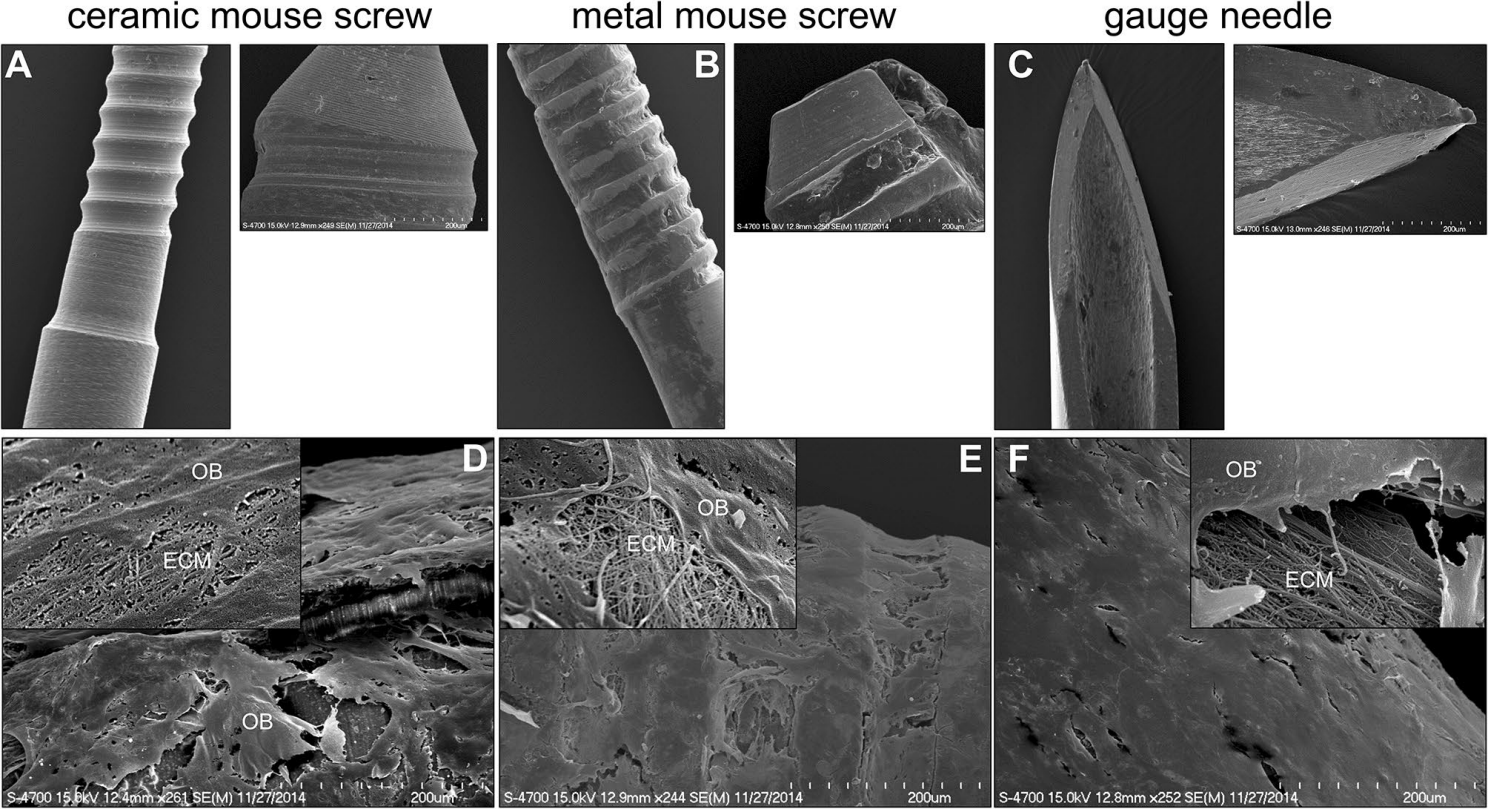
SEM images of the surface of the ceramic MouseScrew (**A**), metal MouseScrew (**B**) and gauge needle (**C**). Magnification 50-fold, small pictures: 250-fold. SEM images of the implant materials with electron microscope imaging of murine primary osteoblasts grown on the surface of the ceramic MouseScrew (**D**), metal MouseScrew (**E**) and gauge needle (**F**) after 25 days of cell culture. Magnification: 250-fold. Inserts: 10,000-fold. *OB* osteoblast, *ECM* extracellular matrix.

In SEM images the surface of the ceramic MouseScrew showed comparable structures to standardized bone implants used in mice such as the gauge needle or the metal MouseScrew (Fig. 3A–C). The attachment and growth of osteoblastic cells (OB) as well as the deposition of extracellular matrix (ECM) were observed on the surface of all three implants (Fig. 3D–F). All materials showed a dense cover of osteoblastic cells embedded in an extracellular matrix on their surfaces.

### Material properties

The mechanical properties of an implant are of fundamental importance when used for intramedullary stabilization, since the biomechanical environment is known to have a direct impact on callus development and maturation. The biomechanical properties of the ceramic device revealed a decreased extension and increased stiffness, whereas the load to failure was comparable to the metal implants (Table 1). Two specimens of each kind were tested, and consistent results with very low variation expected no gain of information by testing more specimen. Therefore, no further testing was performed due to the expenses of the ceramic implant prototypes. The ceramic MouseScrew was the most rigid implant, what has to be taken into account.

**Table 1:** Material properties of the ceramic MouseScrew compared with two commonly used implants, the gauge needle (23G) and the metal MouseScrew (RISystems).

	Measurement	Gauge needle 23G	Metal MouseScrew	Ceramic MouseScrew
Load to failure	1	29.43 N	18.59 N	26.33 N
	2	29.23 N	20.02 N	23.42 N
Extension	1	1.49 mm	1.62 mm	0.67 mm
	2	1.71 mm	1.46 mm	0.66 mm
Stiffness	1	92.59 N/mm	50.49 N/mm	159.84 N/mm
	2	92.79 N/mm	55.43 N/mm	146.60 N/mm

The implant was placed in a material testing machine (Lloyd LRK5) and three point bending testing was performed (n = 2) to determine maximal load [N], extension [mm] and stiffness [N/mm].

### Proof of principle: usage of the ceramic MouseScrew during femur fracture stabilization

#### Surgical technique

Fracture stabilization using the ceramic MouseScrew was performed with eight animals (four female /four male mice). In one female animal the fracture could not be stabilized successfully which can occur occasionally during closed fracture repair. One female animal died due to unknown reasons at day 6 post-surgery and one male animal died after an aggressive attack. Besides that, no other complications could be observed with regard to pain, wound healing, weight bearing or behavior during healing when compared to mice after fracture surgery using metal implants. During MRI analysis, two animals died, most likely due to a prolonged time during establishment of different imaging procedures. In total, no complications that occurred during this study were related to the use of the new ceramic MouseScrew during fracture healing.

#### µCT-imaging of fracture healing progress

**Figure 4:**
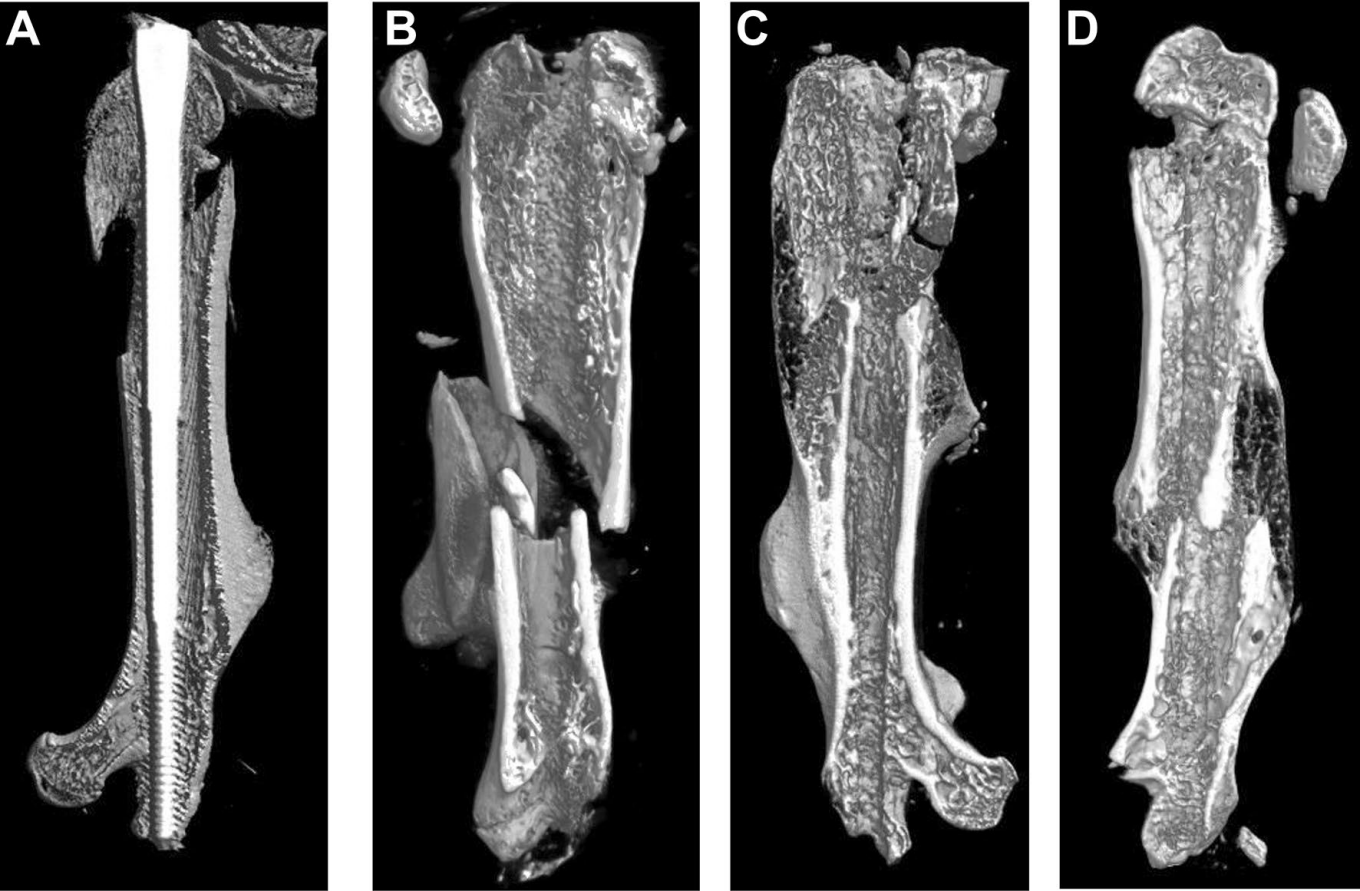
Ex vivo µCT images of fractured femurs of mice stabilized with the ceramic Mouse Screw after 3 days (**A**), with the implant inside the bone), 10 days (**B**) and 4 weeks after fracture (**C**), (**B**,**C**) femurs were scanned without implant). (**D**) Fractured femur, stabilized with a gauge needle, 4 weeks post-surgery as a control example (scanned without implant). Pictures were generated using CTvox 3.3 (Bruker microCT (www.bruker-microct.com)).

Fracture healing outcome in terms of bone consolidation was visualized by µCT of dissected femurs after 3 days, 10 days and 4 weeks using stabilization with the ceramic MouseScrew (Fig. 4A–C). In the first picture (Fig. 4A); the ceramic MouseScrew was left in the bone during image acquisition to visualize the material and density of the device by µCT. In the other images, the implants were removed before image acquisition. An increasing callus formation and bone volume was detectable after 10 days (Fig. 4B) and 4 weeks (Fig. 4C) compared to day 3 (Fig. 4A). When a fractured femur stabilized with a ceramic implant (Fig. 4C) was compared with a fracture stabilized with a gauge needle (Fig. 4D) no obvious differences could be observed. The fracture healing after stabilization with the ceramic MouseScrew was comparable to the fracture healing after stabilization with metal implants.

### MR-imaging of fracture healing progress

**Figure 5:**
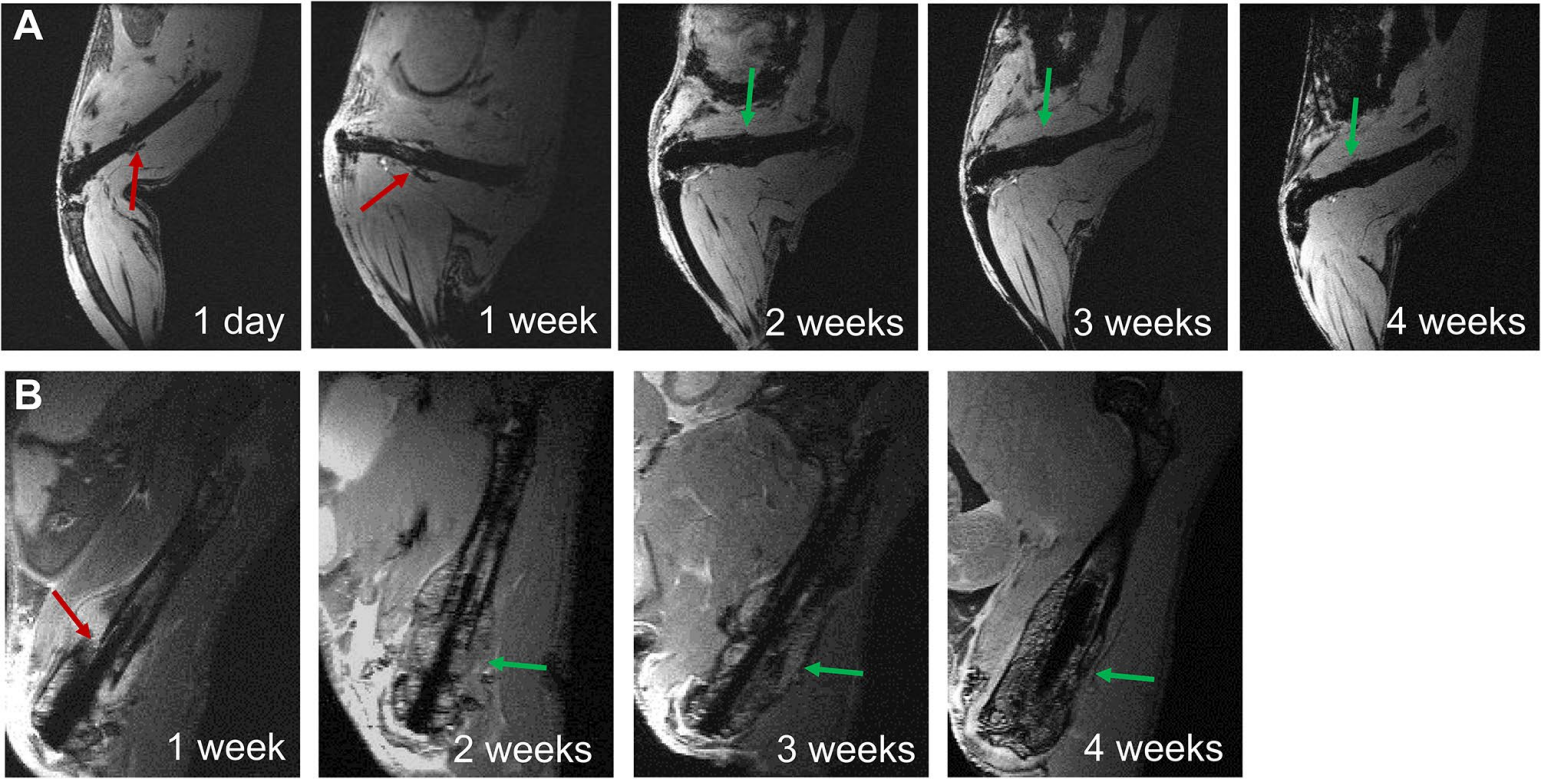
Longitudinal in vivo MRI of fracture healing. 3D T2*-weighted (**A**, FLASH) and 2D T2-weighted images (**B**, RARE) showing the fracture site (red arrow) and callus formation (green arrow) over time course.

In Fig. 5 3D T2* (FLASH, A) and 2D T2 (RARE, B) image series of a fractured femur during healing are shown longitudinally for up to 4 weeks in two animals. In T2*images the fracture site (red arrow) and callus (green arrow) formation could clearly be identified. However, as FLASH images are sensitive to susceptibility differences, clear interfaces between bone tissue and the implant are prohibited. In contrast subcortical and trabecular bone structures could clearly be distinguished from the ceramic MouseScrew in T2-weighted RARE images (Fig. 5B). Detailed bone and callus structures as well as small bony fragments became visible by MRI.

### Quantification of the callus size as a parameter of fracture healing in 3D compared to 2D

**Figure 6:**
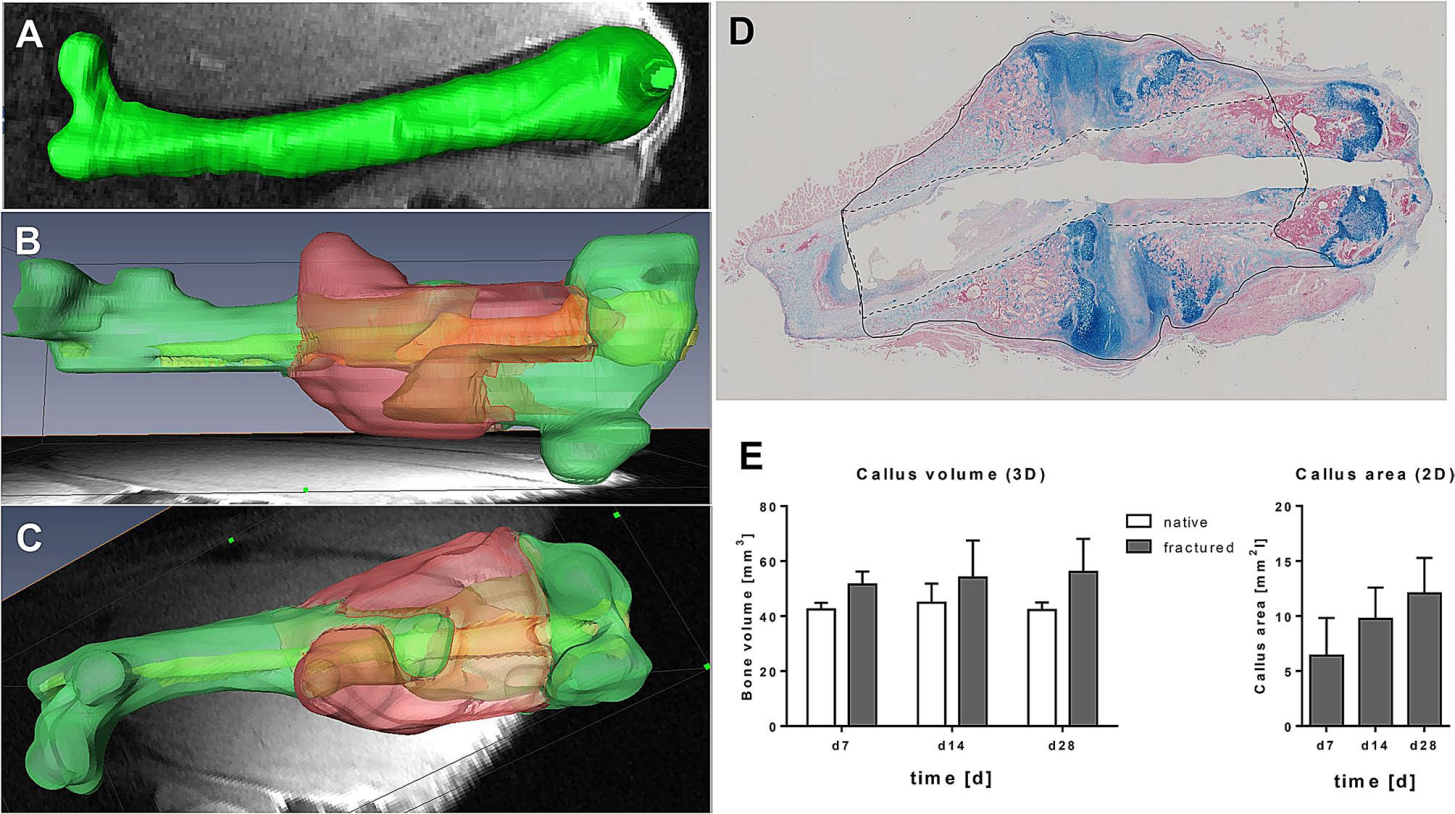
Analysis of the callus size in 2D and 3D as a parameter of the fracture healing outcome. The callus volume was determined by volumetric segmentation of MRI data (mm^3^), (**A**) for comparison, a native femur was also used as a control in 3D analysis; (**B**,**C**) Callus volume shown in red, implant inside the bone in light green, and bone tissue in green, view rotated 90°). (**D**) Paraffin sections (2D) after Alcian blue staining were used to determine the callus area in 2D (mm^2^, callus area is indicated with black line, minus cortical bone and bone marrow/implant channel (dashed line)). (**E**) Quantification of 3D (n = 3) and 2D (n = 4–7) callus measurements, Data are shown as means ± SD. 3D pictures (**A**–**C**) were generated using AMIRA 5.5, ThermoFisher Scientific, www.thermofisher.com); image in (**D**) was generated using Image Pro Plus 7.0, Media Cybernetics Inc, www.mediacy.com.

Callus size and composition are measures of fracture healing progression. We quantified callus formation by volumetric segmentation of a stack of 2D MRT datasets (example shown in Fig. 6A–C, day 14 post-surgery) and histomorphometrical analysis of paraffin slices after Alcian blue staining (Fig. 6D, day 14 post-surgery). As shown in the quantification in Fig. 6E, both, the 2D and 3D quantitative assessments show a clear increase in the callus size over time.

## Discussion

Until now, MRI techniques are not frequently used in fracture healing studies of small animal models due to proportions and artefacts around common metal implants although technical progress helped to overcome resolution problems. In the present study, the development of a novel intramedullary stabilization device made of zirconium dioxide enabled to monitor and quantify the process of fracture healing in vivo and to explore the potential of MRI in a stabilized murine fracture model.

First, we verified that the surface of the new ceramic material showed a roughness that is comparable to approved implants. Surface structure is known to be important for osteoblastic cells to settle and the integration of the implant during healing^28–30^. Furthermore, electron microscopy revealed a multidimensional cell layer with healthy osteoblastic cells embedded in their own extracellular matrix on the surface of the ceramic material comparable to standard metal implants.

Secondly, material properties of the ceramic MouseScrew were tested with regard to mechanical stability in comparison to standard metal implants. The main difference of the ceramic material was a higher stiffness. This might influence the dynamics of bone healing^12,13,31^. Intramedullar stabilization was shown to allow some movement of the bone fragments due to implant material properties that stimulated fracture healing^13^. Therefore, we compared the formation of a bony callus after 4 weeks in both models, ceramic MouseScrew and metal gauge needle using µCT. No obvious differences were observed with regard to faster or delayed healing or callus formation. Stabilization of the bone is known to be one important factor that influences bone healing. Different methods of fixation (plate, nail, fixator) have been used in mice and the healing outcome as well as clinical relevance was compared in the past^19^. Closed fracture and intramedullary stabilization resembles more the human situation with regard to trauma induction, fracture pattern, trauma hematoma and interaction with surrounding tissue (e.g. muscle, blood vessels). It preserves the fracture side without opening of the trauma site, as it has to be performed when using plates or osteotomy models^14,15,19,24^. Some research groups investigating bone structures or bone healing in mice or rats already showed assessment of bone and surrounding tissues with MRI by^4,6,8,9,23,24^. In a recent study by Haffner-Luntzer et al*.*^9^ an external fixator was used in an osteotomy model of mice with ceramic mounting pins that allow MR-Imaging. That study investigated soft tissue, cartilage and interfaces at 11.7 T by high-resolution MRI in comparison to µCT and histomorphology. The authors concluded that the MRI method was reliable to monitor fracture healing. Additionally, it was verified very carefully that the repeated MRI and general anaesthesia had no influence on the fracture healing process.

We showed that by using this ceramic device repeated MRI did allow following fracture healing in individual animal very precisely. As expected, the disadvantage of artefacts of metal implants did not occur. It was clearly possible to identify the implant and the fracture site easily within the femur as well as the growing callus in the course of healing. As a prospective technique to quantify the callus precisely, we introduced volumetric segmentation with e.g. software like AMIRA. With this software, the callus tissue, cortical bone and implant were labeled, visualized and quantified. We were able to show and quantify an increase in callus volume during the course of healing in each animal with low standard variation and comparable to standard 2D histomorphometric analysis of callus sections. Because the total callus tissue often appears not even and circular around the fracture site in this model, a 3D analysis of the whole callus is more reliable and representative than the limited cross sectional analyses of histological tissue sections and therefore is an obvious advantage of the MRI analysis. Due to obvious inter-individual differences of callus growth, following the generation and remodeling of the healing process in an individual animal also represents an additional advantage of magnetic resonance imaging. Supplementary, investigating the time course of bone healing in mice with different time points like we proposed in this study (day 1, 7, 14, 21 and 28) would lead to an animal consumption of at least 8–10 individuals when longitudinal measurements like MRI are used. In contrast to that, histomorphometric analysis with the same time points would require 8–10 animals per time point, meaning 40–50 (= fivefold) mice in total, that have to be sacrificed with additional fivefold more costs for breeding, housing and surgery consumables like implants. It might also be critical for studies with animals carrying genetic modifications that limit fertility or survival. Careful consideration of animal welfare and limited financial funding of research projects would favour the further development of MRI techniques for small animal models.

**Figure 7:**
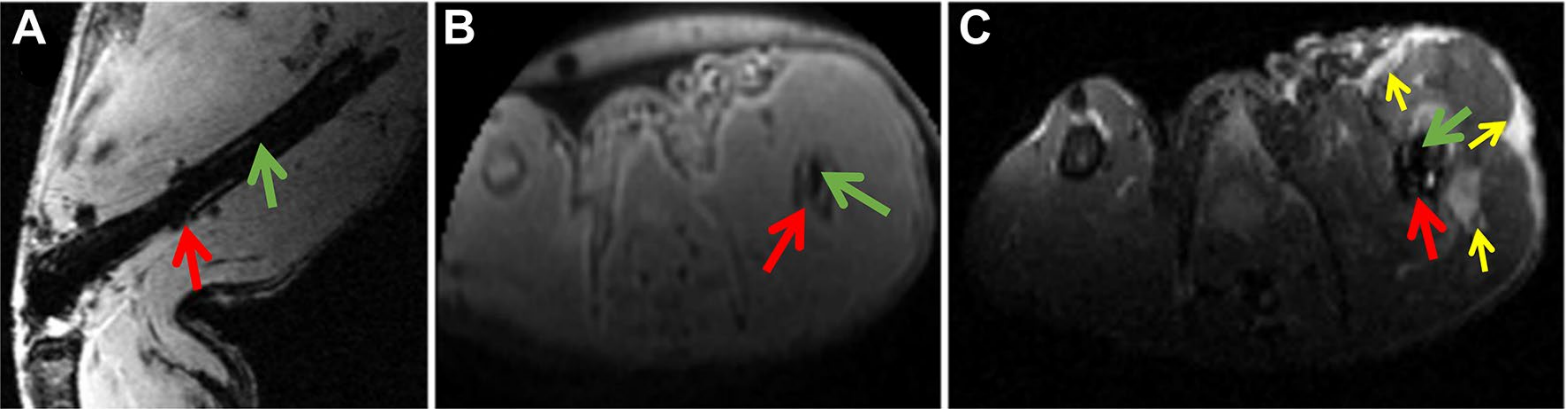
In vivo MRI on day one post fracture showing femur fracture (red arrow), ceramic MouseScrew (green arrow) and edema formation (yellow arrows) in a T2*-weighted (**A**) UTE (**B**) and T2-weighted images (**C**).

As a future perspective, we want to point out that our method of surgery with applying a closed fracture and intramedullary stabilization using a MRI compatible implant will allow further improvement of MRI sequences like FLASH, UTE, RARE or GAG-CEST that will enhance the investigation of the callus tissue in more detail. Additionally, the alterations of the tissue in close neighborhood of the fracture site including skin, muscle tissue and tendons has impact on the healing process that can be evaluated further as well as the healing of the soft tissue after trauma itself. As shown in Fig. 7 1 day post-surgery the tissue shows relevant alterations like edema formation. Initiation and progression of healing at the fracture site might be influenced by the interaction with the surrounding tissue, which is possible to analyze using this implant. Singh et al. proposed a potential for improvement of disease diagnosis, assessment of disease activity and treatment response, and for prognostication by using different MRI techniques that can provide anatomical and functional information in human, but this needed further evaluation^32^. Animal studies will provide important contribution to accelerate this research direction.

### Conclusion

Investigating a novel clinical relevant murine fracture model and stabilization technique to study bone regeneration using an MRI-compatible intramedullary nail, this study clearly demonstrates the benefits of high resolution MRI and volumetric analysis allowing a more detailed differentiation of the callus, bone and tissue composition than we obtained by histomorphometric analysis using tissue sections. Further development of MRI techniques with regard to resolution, differentiation of tissues or imaging methods to label cells or proteins together with compatible implants will pave the way for imaging investigations following dynamic processes like regeneration, remodeling, angiogenesis, inflammation and with that investigation of disorders in bone healing and other musculoskeletal diseases. Finally, it is conceivable that this study may contribute to the development of alternative implants for clinical application providing the possibility to gain more information concerning problems during human fracture healing. Finally yet importantly, the consumption of animals can possibly be lowered drastically with MRI-compatible stabilization systems.

## Materials and methods

### Material properties of the ceramic MouseScrew

#### Surface structure and biocompatibility using scanning electron microscopy (SEM)

The surface of a ceramic MouseScrew, a metal MouseScrew and a gauge needle were analyzed by scanning electron microscopy (SEM). Furthermore, primary osteoblast precursor cells were isolated from calvarias of newborn mice and cultured in presence of l-ascorbate, β-glycerolphosphate and dexametasone for 25 days together with the different implant materials. Thereafter, implant materials were fixed with 4% paraformaldehyde. After critical-point drying, samples were sputter-coated with platinum and the colonized surfaces of the implants were analyzed with a Hitachi S-4700 SEM.

#### Biomechanical characterization of the ceramic MouseScrew

The load, the extension and stiffness of the ceramic MouseScrew (n = 2) were tested in comparison to a gauge needle (23G) (n = 2) and the metal MouseScrew (n = 2) using standard three point bending in a material testing device (Lloyd LK5R) as described in previous work^33^.

### Animal model and study design

All experiments were approved by the state office for nature, environment and consumer protection NRW (LANUV, North-Rhine Westphalia) and were performed according to the protocol approved by the Animal Care and Use Committee of the University Hospital Muenster, Germany (AZ: 84–02.04.2012.A016). All methods were carried out in accordance with relevant guidelines and regulations. Female (6 animals) and male (4 animals) wild type (C57BL/6 genetic background) mice were used at 12 weeks of age for closed, standardized femur shaft fractures. After anesthesia with an intraperitoneal injection of ketamine hydrochloride/xylazine mixture (80 mg/kg and 12 mg/kg body weight) the left knee was bended. A longitudinal incision along the medial side of the patella and patella tendon was applied. After lateral dislocation of the patella the epicondyle was opened using a 0.5 mm centering bit. The guide wire is placed within the bone marrow canal that remains during fracturing using a fracture machine (three point bending). Attached to the guide wire the MouseScrew is inserted into the femur under continuous pressure and rotation until the nail shears of when the sufficient torque is achieved (Manual instructions, RISystem AG, Switzerland). During the operative procedure and directly postoperative a mediolateral X-ray was taken to control the position of the ceramic/metal nail. After correct positioning of the ceramic/metal implant, the wound was closed layer-wise with absorbable suture. Carprofen (4 mg/kg s.c.) was given as an analgesic and then at 24 h intervals as needed. Mobilization and wound healing was evaluated on a daily basis after surgery. After up to 4 weeks after fracture, the mice were euthanized by cervical dislocation. Fractured femurs (and the contralateral femurs as controls) were carefully removed under protection of the callus and used for further clinical assessment.

### MRI

#### Dissected femur

The femur with the implant was subjected to a high resolution ex vivo MRI scanning protocol to assure that the material allows the differentiation of bone and implant without any artifacts. 3D high resolution images (9.4 T on a BioSpec 94/20 small animal MRI system (Bruker Biospin GmbH, Ettlingen, Germnay) equipped with a 0.7 T/m gradient system (BGS-12, Bruker) and ParaVision software version 5.1 (Bruker BioSpin MRI)) were acquired both by FLASH (fast low angle shot) and UTE (ultra-short echo time) sequences. Acquisition parameters for FLASH images were TE/TR, 6.7 ms/30.8 ms; flip angle, 15°; FOV, 25 × 65 × 6 mm^3^; resolution, 39 × 39 × 39 µm^3^; averages,10.For UTE images TE/TR, 20 µs/ 10 ms; FOV, 14 × 14 × 25 mm^3^; resolution, 78 × 78 × 139 µm^3^ were used.

#### In vivo MRI

Mice were anesthetized with 2% isoflurane and were monitored for core body temperature and respiration rate using a MRI-compatible monitoring system (SA Instruments, Stony Brook, NY). The body temperature was kept stable using a warming pad. Imaging was performed using the following sequences and parameters: *3D FLASH* TE/TR, 3.1 ms/20 ms; flip angle, 10°; FOV, 45 × 25 × 26 mm^3^; resolution, 88 × 98 × 197 µm^3^; averages:2. *3D UTE* TE/TR, 20 µs/8 ms; FOV, 24 × 30 × 40 mm^3^; resolution, 188 × 234 × 312 µm^3^. *Axial T2 weighted MSME* image: TE/TR, 10.9 ms/5312.1 ms; FOV, 27.7 × 21.0 mm^2^, resolution, 201 × 201 µm^2^, slice thickness, 0.3 mm. Sequences were optimized for bone tissue contrast.

To analyze the fractured bone volume by segmentation, the MRI image data were acquired using the following parameters: turbo-RARE-2D, TE/TR: 9.3 ms/4.500 ms; FOV, 45 × 25 mm^2^; resolution, 87 × 80 µm^2^; slice thickness, 0.25 mm; RARE-factor, 4; number of averages: 3. As a control the bone volume of native bone was measured by MRI before fracture. After fracture and stabilization with the ceramic MouseScrew the bone volume was measured longitudinally directly 1 day postoperative as well as 1, 2, 3 and 4 weeks post-surgery. Bone volume and callus volume was determined in every section of the MRI dataset and segmentation was carried out using AMIRA, a 3D visualization and analysis software (AMIRA 5.5, ThermoFisher Scientific, Waltham, Massachusetts, U.S.A.). The difference of bone volume between native and fractured bone at the different time points without drill hole and cortex was determined as callus.

### Micro-computer-tomography

Micro-computer tomography (µCT) using a Skyscan 1176 scanner (Bruker, Biospin GmbH, Ettlingen, Germany)) was performed on the femora ex vivo after 3 days, after 10 days and 4 weeks at a resolution of 9 µm using a 0.2 aluminum-filter. After reconstruction, the fractured femur was visualized by manufacturer’s software (NRecon 1.6, Dataviewer 1.5, CTvox 3.3).

### Histology

Anesthesia and fracture surgery were performed as described above. The fracture was stabilized with a gauge needle (23G). 4–9 animals were used per time point. Mice were euthanized after 7, 14 and 28 days. The femora were dissected, fixed and embedded in paraffin. 5 µm thick sagittal sections were stained with Alcian blue. Using digital image analysis, the area of the developed callus tissue was determined histomorphometrically, while the area of the drill hole, bone marrow and the cortex were excluded (software: Image Pro Plus 7.0, Media Cybernetics Inc., Rockville, USA, see Fig. 6D).

### Methods for data management and analysis

Comparison of the data and statistical analysis were carried out using Microsoft Excel (Microsoft Office) and GraphPad Prism version 6.0.7 for Windows, GraphPad Software, San Diego, California USA, www.graphpad.com. Data are shown as mean ± SD.
